# Wahrgenommene Autonomie bei Prostatakarzinom-Langzeitüberlebenden

**DOI:** 10.1007/s00120-026-02854-z

**Published:** 2026-06-05

**Authors:** Cornelia Peter, Ann-Kathrin Christmann, Lilly J. Schmalbrock, Stefan Schiele, Jürgen E. Gschwend, Andreas Dinkel, Kathleen Herkommer

**Affiliations:** 1 Klinik für Urologie, TUM Klinikum Rechts der Isar, Technische Universität München, Ismaninger Straße 22, 81675 München, Deutschland; 2 Klinik für Psychosomatische Medizin und Psychotherapie, TUM Klinikum Rechts der Isar, Technische Universität München, München, Deutschland

**Keywords:** Gesundheitsbezogene Lebensqualität, Subjektives Wohlbefinden, Krebsnachsorge, Radikale Prostatektomie, Psychosoziale Einflussfaktoren, Health-related quality of life, Subjective well-being, Survivorship care, Radical prostatectomy, Psychosocial determinants

## Abstract

**Hintergrund:**

Autonomie ist ein zentrales psychologisches Bedürfnis im höheren Lebensalter und eng mit Lebensqualität, Wohlbefinden sowie funktioneller Leistungsfähigkeit verbunden. Bei Langzeitüberlebenden eines Prostatakarzinoms (PCa) kann das Autonomieerleben durch altersassoziierte Einschränkungen und therapiebedingte Belastungen beeinträchtigt sein.

**Fragestellung:**

Erfassung der wahrgenommenen Autonomie bei PCa-Langzeitüberlebenden und Analyse soziodemographischer, klinischer und psychosozialer Einflussfaktoren.

**Material und Methode:**

Querschnittanalyse im Rahmen des deutschen Forschungsprojekts „Familiäres Prostatakarzinom“. Es wurden 2143 PCa-Langzeitüberlebende (mittleres Alter: 80 Jahre) nach radikaler Prostatektomie eingeschlossen. Die wahrgenommene Autonomie wurde mittels eines Items der Skala „wahrgenommene Autonomie im Alter“ (WAA) erfasst. Es erfolgte eine dichotome Auswertung in „eher autonom“ vs. „eher nicht autonom“. Die Auswertung umfasste soziodemographische, klinische und psychosoziale Parameter.

**Ergebnisse:**

Als „eher autonom“ stuften sich 86 % der Befragten ein. Eine geringere wahrgenommene Autonomie war signifikant mit höherem Alter, niedrigem Bildungsniveau, schlechterer wahrgenommener wirtschaftlicher Situation, aktueller PCa-Therapie, niedrigeren Lebensqualität, niedrigerem subjektivem Wohlbefinden, Progredienzangst und fehlender Internetnutzung assoziiert (alle *p* < 0,001).

**Schlussfolgerungen:**

Die Autonomiewahrnehmung bleibt bei vielen PCa-Patienten, die die Krankheit überlebt haben, langfristig erhalten, ist jedoch durch verschiedene beeinflussbare Faktoren gefährdet. Die Ergebnisse liefern Ansatzpunkte für eine patientenzentrierte, ressourcenorientierte Langzeitversorgung.

**Graphic abstract:**

**Figure:**
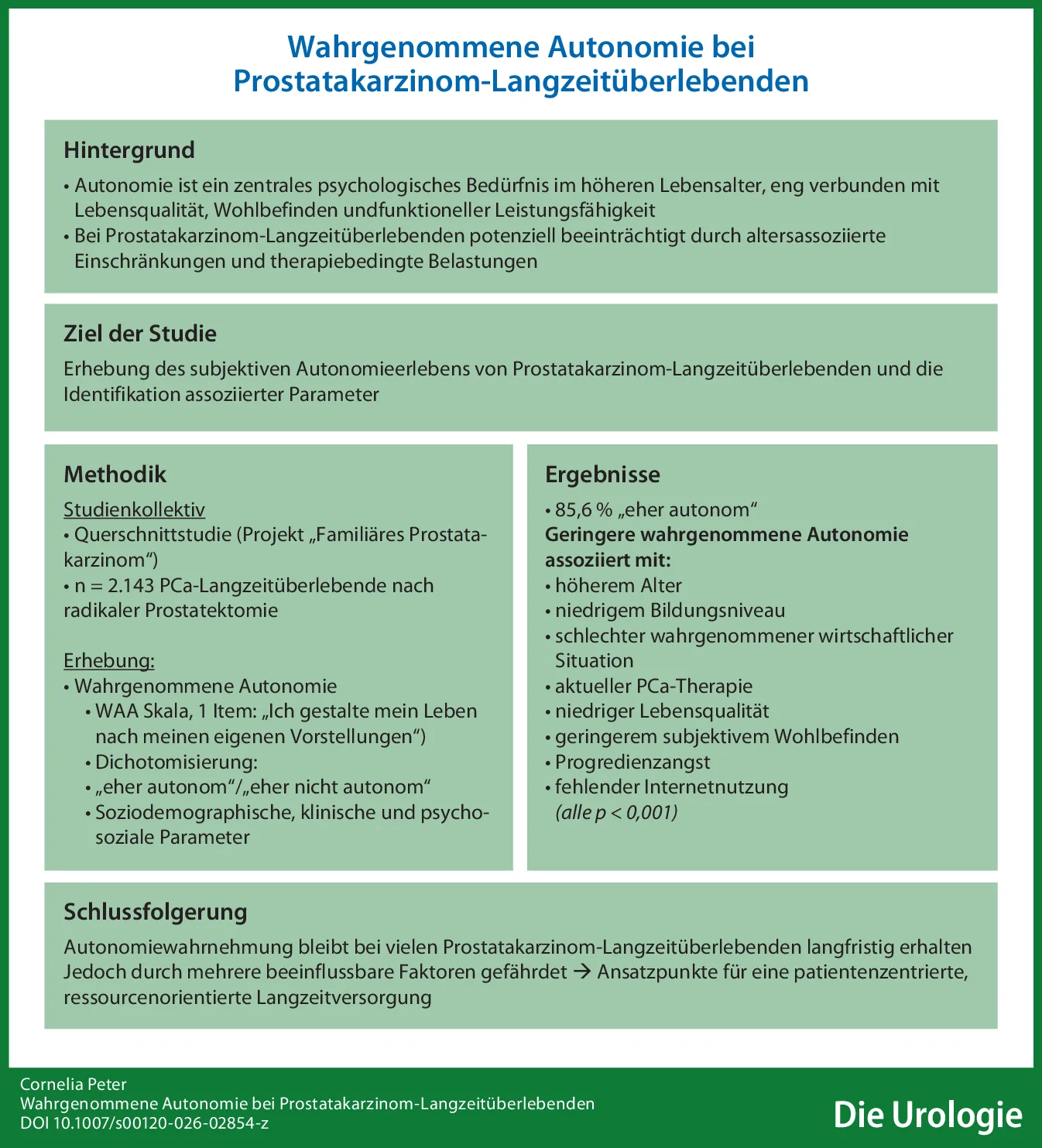

## Abstract (en)

**Background:**

Autonomy is a fundamental psychological need in old age. It is closely linked to quality of life, psychological well-being, and functional capacity. In long-term prostate cancer (PCa) survivors, autonomy may be compromised by age- and treatment-related factors.

**Objective:**

To assess perceived autonomy in long-term PCa survivors and identify sociodemographic, clinical, and psychosocial predictors.

**Methods:**

Cross-sectional analysis of data from the German multicentre project “Familial Prostate Cancer”. A total of 2143 long-term PCa (mean age: 80 years) survivors after radical prostatectomy were included. Perceived autonomy was assessed using an item from the “Perceived Autonomy in Old Age Scale” and dichotomized into “rather autonomous” vs. “rather not autonomous”. Sociodemographic, clinical, and psychosocial factors were analysed.

**Results:**

Overall, 86% of participants perceived themselves as “rather autonomous”. Lower perceived autonomy was significantly associated with higher age, lower education, poorer perceived economic situation, current PCa therapy, reduced quality of life, lower subjective well-being, fear of progression, and lack of daily internet use (all *p* < 0.001).

**Conclusion:**

Although perceived autonomy remains high in many older PCa survivors, it is vulnerable and related to modifiable factors. Findings highlight key targets for a patient-centered, autonomy-supportive approach in long-term uro-oncologic survivorship care.

Autonomie ist ein zentrales psychologisches Grundbedürfnis, das für die Lebensqualität und das Wohlbefinden auch im höheren Lebensalter bedeutsam ist. Bei Langzeitüberlebenden eines Prostatakarzinoms (PCa) kann das Autonomieerleben sowohl durch altersassoziierte Einschränkungen als auch durch therapiebedingte Belastungen beeinträchtigt sein. Um gezielte Maßnahmen zur Erhaltung alltagsbezogener Autonomie in der urologischen Langzeitversorgung ableiten zu können, ist die Analyse relevanter Einflussfaktoren essenziell.

## Hintergrund

Autonomie umfasst mehr als die Fähigkeit zur selbstständigen Bewältigung des Alltags. Sie beschreibt das subjektive Erleben, das eigene Leben entsprechend der persönlichen Vorstellungen gestalten zu können, einschließlich Wahlmöglichkeiten, freiwilliger Entscheidungen sowie der Wahrnehmung von Kontrolle über das eigene Denken, Fühlen und Handeln. Als grundlegendes psychologisches Bedürfnis steht Autonomie in engem Zusammenhang mit Lebensqualität, psychischem Wohlbefinden und funktioneller Leistungsfähigkeit im höheren Lebensalter und gilt als wesentliche Voraussetzung für erfolgreiches Altern [19, 21, 22].

Mit zunehmendem Lebensalter kann die Wahrnehmung der eigenen Autonomie eingeschränkt sein. Altersassoziierte Komorbiditäten sowie funktionelle Einschränkungen, beispielsweise der Mobilität, des Hör- oder Sehvermögens, können die Autonomiewahrnehmung relevant beeinträchtigen [13]. Zusätzlich stellt eine Krebserkrankung eine erhebliche Belastung dar, die durch körperliche Einschränkungen, psychischen Stress, zunehmende Abhängigkeit vom medizinischen Versorgungssystem sowie den Verlust sozialer Rollen mit einer weiteren Reduktion der wahrgenommenen Autonomie einhergehen kann [6, 7].

Prostatakarzinom-Langzeitüberlebende im höheren Lebensalter sind in besonderem Maße betroffen, da sie sowohl altersbedingte Veränderungen als auch krankheits- und therapiebedingte Langzeitfolgen bewältigen müssen. Diese Doppelbelastung stellt einen relevanten Risikofaktor für Einschränkungen der Autonomie dar und macht diese Patientengruppe zu einem besonders wichtigen Kollektiv für die Untersuchung autonomierelevanter Einflussfaktoren.

Autonomie ist dabei nicht mit vollständiger Unabhängigkeit gleichzusetzen. Auch Patienten mit bestehendem Unterstützungsbedarf können ein hohes Maß an Autonomie erleben, sofern sie in medizinische und alltagsbezogene Entscheidungsprozesse einbezogen werden, über reale Wahlmöglichkeiten verfügen und Hilfe selbstbestimmt annehmen. Die Autonomiewahrnehmung ergibt sich aus dem Zusammenspiel individueller Ressourcen, sozialer Unterstützung und struktureller Rahmenbedingungen, die es ermöglichen, trotz funktioneller Einschränkungen aktiv Einfluss auf das eigene Leben zu nehmen [3, 21, 22, 30].

Das PCa ist weltweit die häufigste maligne Tumorerkrankung beim Mann. Bedingt durch die demographische Alterung der Bevölkerung sowie durch Fortschritte in Diagnostik und Therapie steigt die Zahl der PCa-Langzeitüberlebenden kontinuierlich an [12, 17, 32]. In der langfristigen Nachsorge sind therapieassoziierte Nebenwirkungen und funktionelle Beeinträchtigungen häufig beschrieben. Zu den am häufigsten berichteten Folgen zählen Harninkontinenz, Fatigue, sexuelle Funktionsstörungen sowie kognitive Einschränkungen. Zusätzlich werden psychosoziale Belastungen wie Angst, depressive Symptome und eine maladaptive Stressverarbeitung berichtet, die mit einer weiteren Einschränkung der Autonomiewahrnehmung assoziiert sein können [4, 10, 16, 20, 24].

Trotz der wachsenden Zahl an PCa-Langzeitüberlebenden liegen bislang nur begrenzte Daten zur langfristigen Wahrnehmung alltagsbezogener Autonomie sowie zu deren soziodemographischen, klinischen und psychosozialen Einflussfaktoren vor. Ziel der vorliegenden Studie ist es daher, die wahrgenommene Autonomie von PCa-Langzeitüberlebenden im höheren Lebensalter zu untersuchen und relevante Prädiktoren zu identifizieren, um Ansatzpunkte für eine patientenzentrierte urologische Langzeitversorgung herauszuarbeiten.

## Methodik

### Datenbank und Studiendesign

Die vorliegende Analyse ist Teil des nationalen Forschungsprojekts „Familiäres Prostatakarzinom“ des TUM Klinikums Rechts der Isar. Das Forschungsprojekt und die detaillierten Ergebnisse wurden bereits in früheren Publikationen veröffentlicht [11]. Die vorliegende Analyse basiert auf den Daten des Erhebungsjahres 2023. Die Studie wurde von der Ethikkommission des TUM Klinikums Rechts der Isar positiv bewertet (Ethikvotum vom 20.11.2023, Aktenzeichen: 2020-25_3-S-SB). Alle Teilnehmer erteilten eine schriftliche Einwilligung zur Teilnahme und Datenverarbeitung.

### Studienkollektiv

In die Analyse wurden PCa-Langzeitüberlebende eingeschlossen, die den Follow-up-Fragebogen 2023 beantwortet haben. Einschlusskriterien waren eine Primärtherapie mittels radikaler Prostatektomie (RP) sowie die vollständige Beantwortung des Items zur wahrgenommenen Autonomie im Follow-up-Fragebogen 2023, Patienten mit neoadjuvanter Therapie oder Salvage-RP wurden ausgeschlossen. Von den 4996 kontaktierten Patienten konnten 2143 in die abschließende Analyse eingeschlossen werden.

### Erhebungsinstrumente

#### Zielparameter Wahrgenommene Autonomie

Die wahrgenommene Autonomie wurde mit dem Item „Ich gestalte mein Leben nach meinen eigenen Vorstellungen“ der Skala „wahrgenommene Autonomie im Alter“ (WAA) von Schwarzer et al. (2008) erhoben [27]. Wie in zahlreichen quantitativen Studien zur Erfassung von wahrgenommener Autonomie wurde ein Einzelitem der Skala verwendet, um die Vergleichbarkeit der Ergebnisse zu erhöhen [5, 13, 18]. Anhand der Antwortoptionen (vierstufige Likert-Skala) wurden die Patienten in zwei Gruppen eingeteilt: „trifft eher zu“ und „trifft genau zu“ wurden als „eher autonom“ klassifiziert, „trifft kaum zu“ und „trifft nicht zu“ als „eher nicht autonom“ [13].

#### Soziodemographische, klinische und psychosoziale Parameter

Es wurden folgende soziodemographische Parameter (Alter, Partnerschaft, Kinder, Bildungsniveau, wirtschaftliche Situation) und klinische Parameter (Zeit seit RP, familiäre PCa-Anamnese, weitere Krebserkrankungen, aktuelle PCa-Therapie) erhoben. Die psychosozialen Parameter umfassten subjektive Lebensqualität, Benefit Finding, subjektives Wohlbefinden („Happiness“), Progredienzangst sowie Internetnutzung.

Die subjektive Lebensqualität wurde anhand des QL‑2 erhoben, der sich aus den letzten beiden Items des EORTC QLQ-C30 zusammensetzt [1]. Der Rohwert wurde auf eine Skala von 0–100 transformiert. Die Einteilung erfolgte in zwei Gruppen: niedrige gesundheitsbezogene Lebensqualität (< 70) und hohe gesundheitsbezogene Lebensqualität (≥ 70) [8].

Benefit Finding wurde anhand eines Einzelitems der Benefit-Finding-Skala (BFS) erfasst: „Meine Prostatakrebserkrankung hat dazu beigetragen, meinem Leben Sinn zu verleihen und andere Schwerpunkte zu setzen.“ Die Einteilung erfolgte in zwei Gruppen: hohes Benefit Finding (mittelmäßig, ziemlich, sehr) und niedriges Benefit Finding (manchmal, gar nicht) [2].

Subjektives Wohlbefinden („Happiness“) wurde mithilfe eines modifizierten Einzelitems erfasst: „Wie häufig haben Sie sich im vergangenen Monat glücklich gefühlt?“ Die Antworten wurden in zwei Gruppen eingeteilt: „hohes Wohlbefinden“ (oft, die ganze Zeit) und „niedriges Wohlbefinden“ (gar nicht, manchmal) [23].

Zur Erfassung der Progredienzangst wurde die Kurzform des Progredienzangstfragebogens (PAF-KF) verwendet. Der Fragebogen umfasst 12 Items, die auf einer fünfstufigen Skala (1–5) beurteilt werden, mit einem Wertebereich von 12–60. Ein Summenwert von mindestens 34 wird als Hinweis auf eine klinisch relevante Progredienzangst gewertet [15, 25].

### Statistische Analyse

Zur deskriptiven Analyse der Stichprobe wurde für kategoriale Variablen die absolute sowie die relative Häufigkeit und für stetige Variablen der Mittelwert (M) sowie die Standardabweichung (SD) angegeben. Zur Verbesserung der Vergleichbarkeit und klinischen Interpretierbarkeit wurden zentrale Variablen anhand etablierter Cut-offs dichotomisiert. Soziodemographische, klinische und psychosoziale Variablen wurden zwischen den Patienten, die „eher autonom“ und denen die „eher nicht autonom“ waren, mittels χ^2^-Test verglichen. Die statistischen Analysen wurden mit SAS, Version 9.4, durchgeführt. Ein Signifikanzniveau von *p* < 0,05 wurde als statistisch signifikant angesehen.

## Ergebnisse

Die durchschnittliche Zeit seit der RP betrug 19 (± 3,7) Jahre. Das durchschnittliche Alter der Patienten zum Zeitpunkt der Befragung betrug 80 (± 6,5) Jahre. Rund 60 % der Befragten waren 80 Jahre oder älter. Ein Großteil der Patienten lebte in einer festen Partnerschaft (84 %) und hatte mindestens ein Kind (89 %). Hinsichtlich der Wohnsituation lebte der überwiegende Teil der Befragten gemeinsam mit der Partnerin/Partner zur Miete oder im Eigenheim (72 %). 18 % der Patienten wohnten allein zur Miete oder im Eigenheim, während 8 % mit Familienangehörigen zusammenlebten. In Bezug auf das Bildungsniveau wiesen 47 % ein hohes, 16 % ein mittleres und 37 % ein niedriges Niveau auf. 75 % der Patienten schätzten ihre wirtschaftliche Situation als (sehr) gut ein. Die Mehrheit der Patienten (92 %) berichtete, sich in regelmäßiger Nachsorge bezüglich des PCa zu befinden. Zum Befragungszeitpunkt erhielten 12 % der Patienten eine PCa-Therapie.

Progredienzangst wurde von 7 % der Patienten angegeben. Eine hohe Lebensqualität wurde von 53 % angegeben, ein hohes Maß an subjektivem Wohlbefinden („Happiness“) von 69 %. Die Hälfte der Patienten (49 %) gab an, das Internet täglich zu nutzen (Tab. 1).

**Tab. 1:** Soziodemographische, klinische und psychosoziale Parameter der Studienpopulation (*n* = 2143)

**Soziodemographische Parameter**	**MW (±SD)**	***n***	**%**
*Alter bei Befragung*** (**Jahre, MW [±SD])	80,5 (± 6,5)	–	–
*Partnerschaft*			
Ja	–	1777	83,9
Nein	–	342	16,1
*Kinder*			
Ja	–	1887	88,9
Nein	–	235	11,1
*Bildungsniveau*			
Niedrig	–	732	37,1
Mittel	–	317	16,1
Hoch	–	922	46,8
*Wirtschaftliche Situation*			
(Sehr) gut	–	1479	75,3
Zufriedenstellend/weniger gut/schlecht	–	485	24,7
*Wohnsituation*			
Lebe allein zur Miete/im Eigenheim	–	374	18,2
Lebe mit PartnerIn zur Miete/im Eigenheim	–	1487	72,2
Lebe mit Familienangehörigen zur Miete/im Eigenheim	–	172	8,4
**Klinische Parameter**	**MW (±SD)**	***n***	**%**
*Zeit seit RP*** (**Jahre, MW [±SD])	19,2 (± 3,7)	–	–
*Positive PCa-Familienanamnese*			
Ja	–	902	42,1
Nein	–	1241	57,9
*Weitere Krebserkrankung*			
Ja	–	291	13,6
Nein	–	1852	86,4
*Therapie zum Befragungszeitpunkt*			
Ja	–	261	12,2
Nein	–	1880	87,8
**Psychosoziale Parameter**	–	***n***	**%**
*Lebensqualität*			
Hoch (≥ 70)	–	1116	52,5
Niedrig (< 70)	–	1008	47,5
*Benefit Finding*			
Hoch (mittelmäßig/ziemlich/sehr)	–	1073	50,5
Niedrig (manchmal/gar nicht)	–	1050	49,5
„*Happiness*“			
Hoch (oft/die ganze Zeit)	–	1465	68,6
Niedrig (manchmal/gar nicht)	–	671	31,4
*Progredienzangst*			
Ja (≥ 34)	–	129	6,8
Nein (< 34)	–	1763	93,2
*Internetnutzung täglich*			
Ja	–	881	49,4
Nein	–	903	50,6

*MW* Mittelwert, *PCa* Prostatakarzinom, *SD* „standard deviation“

### Zielparameter wahrgenommene Autonomie

**Abb. 1:**
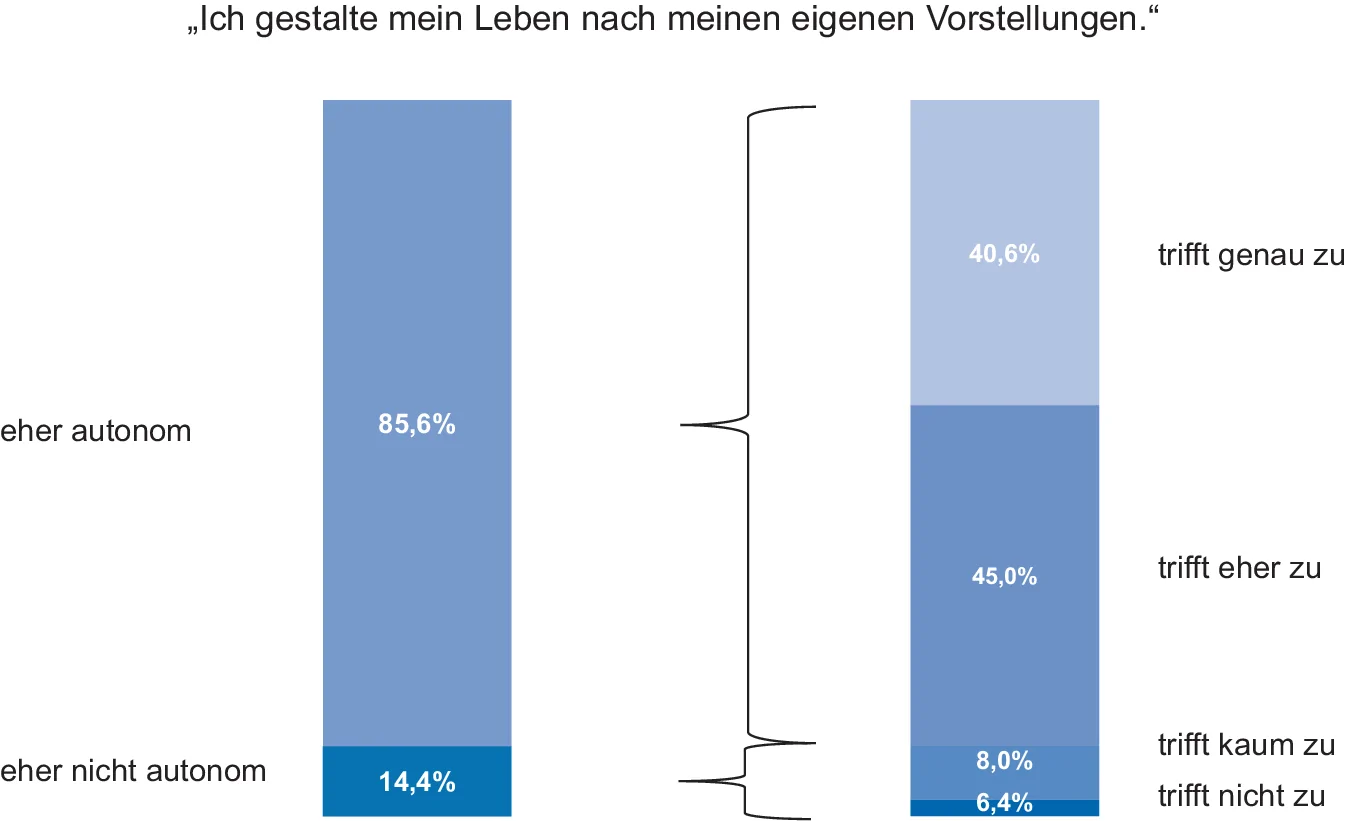
Verteilung der Patienten auf die Gruppen „eher autonom“ und „eher nicht autonom“ in Relation zu den gegebenen Antworten auf die erhobene Frage

Der Zielparameter wahrgenommene Autonomie zeigte folgende Verteilung der Antworten: 41 % der Patienten wählten „trifft genau zu“, 45 % „trifft eher zu“, 8 % „trifft kaum zu“ und 6 % „trifft nicht zu“. Damit nahmen sich 86 % der Patienten als „eher autonom“ und 14 % als „eher nicht autonom“ wahr (Abb. 1).

### Einflussfaktoren auf die wahrgenommene Autonomie

Höheres Alter, niedrigeres Bildungsniveau, Wohnsituation (Zusammenleben mit Familienangehörigen) sowie eine subjektiv schlechter eingeschätzte wirtschaftliche Situation waren signifikant mit einer geringeren wahrgenommenen Autonomie assoziiert (alle *p* ≤ 0,001). Von den berücksichtigten klinischen Parametern war nur eine laufende PCa-Therapie signifikant mit einer geringeren wahrgenommenen Autonomie assoziiert (*p* = 0,004). Bei den psychosozialen Parametern zeigte sich, dass alle bis auf Benefit finding mit einer geringeren wahrgenommenen Autonomie assoziiert waren. Patienten mit niedriger Lebensqualität, geringerem subjektivem Wohlbefinden („Happiness“), erhöhter Progredienzangst sowie mit seltener Internetnutzung (nicht täglich), berichteten häufiger eine geringere wahrgenommene Autonomie (alle *p* < 0,001; Tab. 2).

**Tab. 2:** Verteilung der soziodemographischen, klinischen und psychosozialen Parameter in den Gruppen „eher autonom“ vs. „eher nicht autonom“ nach (*n* = 2143)

	Eher autonom (%)	Eher nicht autonom (%)	*p*-Wert
**Soziodemographische Parameter**			
*Alter bei Befragung (Jahre)*	–	–	*<* *0,001*
< 80	89,4	10,6	–
≥ 80	82,0	17,1	–
*Partnerschaft*	–	–	0,105
Ja	85,3	14,7	–
Nein	88,6	11,4	–
*Kinder*	–	–	0,135
Ja	85,3	14,7	–
Nein	88,9	11,1B	–
*Bildungsniveau*	–	–	*<* *0,001*
Niedrig	80,9	19,1	–
Mittel	87,7	12,3	–
Hoch	88,9	11,1	–
*Subjektive wirtschaftliche Situation*	–	–	*<* *0,001*
(Sehr) gut	87,6	12,4	–
Zufriedenstellend/weniger gut/schlecht	81,2	18,8	–
*Wohnsituation*	–	–	*0,001*
Lebe allein zur Miete/im Eigenheim	90,6	9,4	–
Lebe mit PartnerIn zur Miete/im Eigenheim	85,7	14,3	–
Lebe mit Familienangehörigen zur Miete/im Eigenheim	79,1	20,9	–
**Klinische Parameter**			
*Positive PCa-Familienanamnese*	–	–	0,185
Ja	86,8	13,2	–
Nein	84,8	15,2	–
*Weitere Krebserkrankung*	–	–	0,743
Ja	86,3	13,8	–
Nein	85,5	14,5	–
*Therapie zum Befragungszeitpunkt*	–	–	*0,004*
Ja	80,0	20,3	–
Nein	86,4	13,6	–
**Psychosoziale Parameter**			
*Lebensqualität*	–	–	*<* *0,001*
Hoch (≥ 70)	90,7	9,3	–
Niedrig (< 70)	80,3	19,7	–
*Benefit Finding*	–	–	0,053
Hoch (mittelmäßig/ziemlich/sehr)	87,1	12,9	–
Niedrig (manchmal/gar nicht)	84,2	15,8	–
„*Happiness*“	–	–	*<* *0,001*
Hoch (oft/die ganze Zeit)	88,7	11,3	–
Niedrig (manchmal/gar nicht)	79,0	21,0	–
*Progredienzangst*	–	–	*<* *0,001*
Ja (≥ 34)	71,3	28,7	–
Nein (< 34)	87,0	13,0	–
*Internetnutzung täglich*	–	–	*<* *0,001*
Ja	90,2	9,8	–
Nein	83,1	16,9	–

*PCa* Prostatakarzinom

## Diskussion

In der vorliegenden Studie wurde die wahrgenommene Autonomie von 2143 PCa-Langzeitüberlebenden nach radikaler Prostatektomie untersucht. Die Mehrheit der Patienten (86 %) nahm sich als „eher autonom“ wahr. Die in unserer Studie beobachtete hohe Prävalenz wahrgenommener Autonomie entspricht den Ergebnissen anderer deutscher Studien, die ebenfalls ein Einzelitem der WAA verwendeten und zeigen, dass die Mehrheit älterer Menschen angibt, ihr Leben nach eigenen Vorstellungen gestalten zu können [13, 18]. 15 % der Patienten berichteten über eine niedrige wahrgenommene Autonomie, was auf einen potenziellen Unterstützungsbedarf in diesem Bereich hinweist.

### Soziodemographische Einflussfaktoren

Ein höheres Alter war signifikant mit einer niedrigeren wahrgenommenen Autonomie assoziiert. Funktionelle Einschränkungen, Mobilitätsverluste und zunehmende gesundheitliche Belastungen im höheren Lebensalter gelten als Faktoren, die das Autonomieerleben beeinträchtigen können [9, 13]. Auch ein niedrigeres Bildungsniveau stand signifikant mit einer geringeren wahrgenommenen Autonomie in Zusammenhang. Bildung ist häufig mit höherer Gesundheitskompetenz, besseren Bewältigungsressourcen und gesteigerter Selbststeuerung assoziiert, was die selbstständige Alltagsgestaltung unterstützen kann [26]. Zudem war eine als schlechter eingeschätzte wirtschaftliche Situation signifikant mit einer niedrigeren wahrgenommenen Autonomie assoziiert. Finanzielle Ressourcen beeinflussen maßgeblich, in welchem Umfang Unterstützungsangebote genutzt und individuelle Handlungsspielräume aufrechterhalten werden können. Ein signifikanter Zusammenhang zeigte sich für die Wohnsituation. Personen, die allein leben, berichteten eine höhere wahrgenommene Autonomie als Personen in Mehrpersonenhaushalten. In größeren Haushalten können Abstimmungs- und Koordinationsprozesse die individuellen Entscheidungsspielräume potenziell stärker begrenzen [31].

### Klinische Einflussfaktoren

Unter den klinischen Parametern war lediglich eine zum Befragungszeitpunkt laufende PCa-Therapie signifikant mit einer niedrigeren wahrgenommenen Autonomie assoziiert. Therapien wie die Androgendeprivationstherapie können sowohl physische Einschränkungen als auch psychische Belastungen mit sich bringen [6]. Diese Einschränkungen können wiederum die Handlungsfähigkeit und Entscheidungsfreiheit beeinträchtigen, die wesentliche Aspekte des Autonomieerlebens darstellen. In der Folge kann es beispielsweise zum Verlust vertrauter Rollen im sozialen, familiären oder beruflichen Umfeld kommen.

### Psychosoziale Einflussfaktoren

Eine niedrigere subjektive Lebensqualität war signifikant mit einer niedrigeren wahrgenommenen Autonomie assoziiert. Da das Gefühl der Selbstbestimmung zentral zur Bewertung der eigenen Lebenssituation beiträgt, stehen Lebensqualität und Autonomie in enger Wechselwirkung [20]. Auch ein geringeres subjektives Wohlbefinden („Happiness“) war signifikant mit einer niedrigeren wahrgenommenen Autonomie verbunden. Glückserleben ist eng mit Selbstwirksamkeit, Kontrolle und aktiver Alltagsgestaltung verknüpft. Entsprechend können Einschränkungen in diesen Bereichen das psychische Wohlbefinden beeinträchtigen [29]. Patienten mit einer klinisch relevanten Progredienzangst berichteten ebenfalls signifikant häufiger über eine niedrigere wahrgenommene Autonomie. Die Angst vor Krankheitsfortschritt kann zu übervorsichtiger Alltagsgestaltung, Rückzug oder Entscheidungsunsicherheit führen – Faktoren, die dem Gefühl von Selbstbestimmung entgegenwirken [15, 16]. Ein Zusammenhang bestand ferner zur täglichen Internetnutzung, die signifikant mit höherer wahrgenommener Autonomie assoziiert war. Digitale Teilhabe ermöglicht Informationsbeschaffung, soziale Kommunikation und die Organisation des Alltags und erleichtert somit viele Lebensbereiche, auch bei körperlichen Einschränkungen [18, 28]. Die Assoziation zwischen Internetnutzung und höherer Autonomie unterstreicht die Bedeutung digitaler Teilhabe, die in dieser älteren Kohorte jedoch limitiert sein kann.

### Limitationen

Die Studie profitiert von einer großen und bundesweit rekrutierten homogenen Stichprobe, wodurch eine hohe Repräsentativität erzielt wird. Gleichzeitig begrenzt diese Homogenität die Generalisierbarkeit auf andere Tumorentitäten oder Behandlungsformen. Ein möglicher Selektionsbias ergibt sich aus dem erhöhten Anteil familiär belasteten Patienten (ca. 25 % vs. ca. 15–20 % in der Allgemeinbevölkerung [14]), was die Übertragbarkeit der Ergebnisse einschränken könnte.

## Ausblick

Die Ergebnisse der Studie zeigen, dass die wahrgenommene Autonomie auch im hohen Alter und viele Jahre nach einer Prostatakarzinomdiagnose erhalten bleiben kann, jedoch durch eine Vielzahl soziodemographischer, klinischer und psychosozialer Faktoren beeinflusst wird. Die Ergebnisse zur wahrgenommenen Autonomie lassen sich in die bestehende Literatur einordnen, in der Autonomie im höheren Lebensalter als zentrale Ressource erfolgreichen Alterns sowie als bedeutender Prädiktor für Lebensqualität und psychisches Wohlbefinden beschrieben wird [20, 27]. Für die Praxis sind diese Erkenntnisse besonders relevant, um die individuelle Förderung in der Langzeitnachsorge zu verbessern.

Gezielte Maßnahmen wie niedrigschwellige und verständliche Informationsangebote, psychosoziale Unterstützung, die Förderung der digitalen Teilhabe sowie eine bedarfsgerechte Nachsorgeplanung können dabei helfen, die Autonomie gefährdeter Patientengruppen zu erhalten oder zu verbessern.

## Fazit für die Praxis

- Trotz hohen Lebensalters zeigen Prostatakarzinom (PCa)-Langzeitüberlebende ein hohes Maß an Autonomie.
- Die wahrgenommene Autonomie bleibt bei vielen Betroffenen langfristig erhalten; sie sollte jedoch regelmäßig im Patientengespräch erfasst werden, um vulnerable Patientengruppen frühzeitig zu identifizieren.
- Eine aktuelle PCa-Therapie sowie Progredienzangst stellen Risikofaktoren für eingeschränkte Autonomie dar. Eine gezielte Patientenedukation und psychosoziale Unterstützung sind daher essenziell.
- Digitale Teilhabe fördert das Autonomieerleben: Niederschwellige Informationsvermittlung (z. B. zu Patientenportalen, Selbstmanagement-Apps oder telemedizinischen Angeboten) kann die Selbstständigkeit stärken. Patienten sollten daher aktiv auf verständliche und niedrigschwellige digitale Unterstützungsangebote hingewiesen werden.

## Data Availability

Alle dieser Arbeit zugrunde liegenden Daten sind in diesem Artikel enthalten.
